# Innovation in observation: a vision for early outbreak detection

**DOI:** 10.3134/ehtj.10.006

**Published:** 2010-05-20

**Authors:** NH Fefferman, EN Naumova

**Affiliations:** 1 Department of Ecology, Evolution and Natural Resources, Rutgers University, New Brunswick, NJ, USA; 2 Department of Civil and Environmental Engineering, Tufts University School of Engineering, Medford, MA, USA

## Abstract

The emergence of new infections and resurgence of old ones—health threats stemming from environmental contamination or purposeful acts of bioterrorism—call for a worldwide effort in improving early outbreak detection, with the goal of ameliorating current and future risks. In some cases, the problem of outbreak detection is logistically straightforward and mathematically easy: a single case of a disease of great concern can constitute an outbreak. However, for the vast majority of maladies, a simple analytical solution does not exist. Furthermore, each step in developing reliable, sensitive, effective surveillance systems demonstrates enormous complexities in the transmission, manifestation, detection, and control of emerging health threats. In this communication, we explore potential future innovations in early outbreak detection systems that can overcome the pitfalls of current surveillance. We believe that modern advances in assembling data, techniques for collating and processing information, and technology that enables integrated analysis will facilitate a new paradigm in outbreak definition and detection. We anticipate that moving forward in this direction will provide the highly desired sensitivity and specificity in early detection required to meet the emerging challenges of global disease surveillance.

We cannot create observers by saying ‘observe,’ but by giving them the power and the means for this observation and these means are procured through education of the senses.

Maria Montessori

## Introduction: Three main challenges in outbreak detection

Failure of biosurveillance systems to detect incipient outbreaks at the earliest stages of spreading infection can lead to otherwise avoidable increases in the incidence of and mortality from a disease, whether that disease results from environmental contamination, exposure to zoonotic pathogens, or purposeful acts of bioterrorism. Many different mathematical and statistical techniques have therefore been proposed^1, 2^ to analyse incoming disease incidence reports and detect a signal that is distinct from the unavoidable noise as early as possible after the onset of any event. However, the possibilities are far from exhausted and we have not yet developed a demonstrably reliable early detection method. The availability of better methods will invariably lead to enhanced biosecurity and help government, policy makers, and safety professionals in general to provide more effective strategies to ensure public health and safety.

For some diseases of greatest concern, the problem of outbreak detection is mathematically easy: a single case can constitute an outbreak (for example, smallpox), and for diseases for which this is the case, our efforts are geared towards early biological detection and identification of the single case, rather than mathematically or statistically identifying an impending epidemic. However, for the vast majority of diseases, including those responsible for frequent and/or periodic epidemics and severe mortality (for example, influenza, which claims B36 000 lives a year in the United States under normal, non-epidemic conditions^3^, our concern in biosurveillance and outbreak detection is rather focused on finding ways to detect unusual patterns of disease incidence soon after they begin to break from what is expected, typical, or endemic. This brings us to our first challenge in modern biosurveillance: the definition of ‘an outbreak’. A large part of the effort for a number of diseases is already spent in making the decision of what constitutes an unusual pattern.^4^ Most modern disease surveillance systems rely on static thresholds for outbreak detection. Outbreaks are declared once a sufficient number of new cases are reported over a certain period of time, within the confines of a certain location. The determination of these thresholds (that is, how many cases, over how long a period of time, within how much space) relies on historical incidence data to set an expected baseline for ‘normal’ conditions. This approach is instantly problematic when the disease of concern has never been observed within the population at risk, which means that no baseline can be established; however, it also poses other challenges. No two outbreaks are ever identical, even once corrections are made for population size, demography, and preexisting protective immunity. Similarly, endemic (or non-outbreak) disease occurrence can fluctuate for a variety of reasons (for example, known seasonal patterns). This means that there are large challenges involved in both determining what is normal and determining what is unusual. Methods from the fields of operations research and traditional statistics have helped somewhat, although the usual goal in operations research is to detect an event exceeding the ‘3 sigma’ threshold of detection, based on the likelihood and frequency of the occurrence.^5–7^ In biosurveillance, however, the likelihood and frequency of an event of interest are not actually determinants of concern. Even if local outbreaks of severe acute respiratory syndrome or Ebola haemorrhagic fever became frequent, and even expected, it would still be a logical requirement of any biosurveillance system to detect these events as soon as possible.

Some more sensitive methods (compared with a fixed threshold approach) for outbreak detection have proposed the use of adaptive thresholds, incorporating information on patterns in disease incidence over some past units of time, as well as the current scenario, to determine whether observed patterns of incidence signal an incipient outbreak.^5, 8^ These methods, however, involve complications of their own: not only are historical data needed for both outbreak and nonoutbreak conditions of the past against which current trends must be compared, but also the trajectories must be considered. This implies a requirement for much greater richness in historical data. If not all possible paths for the spread of infection have occurred within the period for which historical records are used for comparison, these methods may be unable to classify an outbreak simply because the trajectory of increased incidence is unfamiliar. Naturally, the number of different possible trajectories is much larger than the number of different current threshold scenarios, requiring a much longer and richer historical data set before a meaningful baseline can be set than would be required to detect outbreaks were all trajectories naturally identical. Hybrid systems, separately using both current threshold violations and adaptive trajectory responses, have solved some of these problems. (For example, when shifts in health-care utilization during epidemics and major public events are expected, an integrated network model of epidemiological data streams might have the potential to improve localized outbreak detection.^9^

However, the definition of an outbreak is not the only major stumbling block to implementing a practical biosurveillance system to support the efforts of public health officials. Even if we assume that, for each disease, we have a very good definition of what constitutes normal, endemic levels and what should constitute an outbreak worthy of our attention, various incredibly complex challenges remain. This brings us to our second challenge: heterogeneity in the sources of data. Diversity in incoming data provides a multifaceted source of information, and also challenges any system by providing variation in the timing and scale of reported incoming values. For example, daily, weekly, and monthly counts of cases may be reported as signals of the same disease, covering the same population. As we develop truly multifaceted sources of input into detection systems, involving data from such varied sources as medication sales, hospital admissions, doctors visits, web queries, and school or office absenteeism, integrating signals requires compensation for the existence of single cases contributing to multiple signals, not always simultaneously. Furthermore, not all of these signals will have equal significance to outbreak detection, nor is it reasonable to expect that actual incidence of infection will be the only factor affecting their dynamics.

Finally, even if the difficulties in defining an outbreak and integrating and appropriately weighing the various sources of incoming surveillance data can be overcome, there still remains our third challenge: timely detection of outbreaks. Different diseases will require different lead times in order to allow officials to mount effective and appropriate public health response measures. However, common to all possible outbreaks is the desire for reliable outbreak detection as early as can be managed. Naturally, making the system reliable is one of the key constraints against providing the earliest possible warning of a coming problem. A system that routinely issues false alarms is potentially even more harmful than a system that fails to notice an outbreak until it is already past its epidemic peak. Finding an appropriate balance between sensitivity and specificity is the last great challenge to the creation of effective measures in outbreak detection.

## Overview of traditional and upcoming data sources and data streams

Worldwide disease surveillance systems are historically supported by a reliable network of hospitals, outpatient clinics, and diagnostic facilities, and are operated in cooperation with local and regional public health institutions. Overall, the established infrastructure facilitates consistent improvement in data quality, essential for reliable monitoring of well-controlled diseases. Most data-monitoring systems that form the basis for many national surveillance programmes strive to ensure a complete and comprehensive coverage of the general population. Typically, medical and social care facilities, diagnostic services networks, schools, universities, workplaces, and correctional facilities constitute the core of data sources for universal data repositories (see Table 1 for more examples). Their ability to reflect how an outbreak progresses varies substantially with different data sources, and the selection of a single source to provide reliable early outbreak detection is problematic. For example, highly specialized and disease-oriented systems are likely to detect a change in a disease of interest and to offer high-quality data on pathogen specifications by using molecular genetic typing.^10^ However, such systems might not be flexible enough to track a novel pathogen or to collect and process new information associated with an outbreak in a timely manner and at reasonable cost. On the other hand, systems focusing on a search for an aberration in ‘noisy’ environments, for example, syndromic surveillance, might detect a change at an appropriate time, but fail to specify the cause.^11^

**Table 1:** Examples of data sources, streams, curators, and supplementary information supporting surveillance systems

*Data sources*	*Data streams*	*Data curators*	*Auxiliary data*
Medical care facilities (hospitals,	Death records	Public health institutions	Demographic profiles
health-care and rehabilitation centres,	Chief complains for emergency	Local and regional	Vaccination coverage records
ambulatory clinics, drug dispensaries)	room visits	departments of vital statistics	Calendars of social activities
Diagnostic testing facilities	Medical service and equipment use	National health statistics	Environmental samples
(laboratories, mobile diagnostic units)	records	institutions	Population migration and
Social-care facilities (day care centres,	Prescription records	Insurance industry	displacement patterns
assisted living and nursing homes,	Insurance claims	Pharmaceutical industry	Land use, climate, and
hospice services)	Laboratory tests	Governmental and non-	meteorological information
Correctional facilities (jails, prisons)	Pharmaceutical sale	governmental organizations	Domestic animals and wildlife
Schools	records	Academic institutions	surveillance data
Work places	Absenteeism reports		
Locations of intentional screening	Hotline calls		
	PDA records		
	Website queries		
	Media news clips		
	Forensic records		

Each data source provides one or more data streams that differ by a number of characteristics important for early outbreak detection. Efforts to better understand the magnitude and severity of emerging and reemerging health threats force the search for various proxies to diseases. A rapid incorporation of novel data streams into disease surveillance systems has demonstrated the potential of an increasing information exchange.^12^–^15^ The list of prospective candi dates for disease monitoring is growing and includes not only traditional hospitalization records and laboratoryconfirmed tests but also the data streams driven by novel information technologies. Personal digital assistant-based records collected directly from an outbreak investigation,^16^ hits and queries targeting specific websites (http://www.google.org/flutrends/),^17^ satellite imagery,^18^ and searches for media and news items are examples of these new data streams (http://www.healthmap.org/about.php).

The inadequacy of existing infrastructure to provide early warning even with a combination of standard and new health measures is a problem that cannot be easily resolved. A blend of new and traditional data streams offers a wide range of measurable health and disease outcomes with highly desirable characteristics in specificity; however, the breadth and diversity of new measures and their composites come with the price of presenting new challenges in data processing. Each additional data stream has its own characteristics, including timing, sensitivity and specificity, accuracy, population coverage, and overlap with other sources. Data streams that originate from various sources might have differences in their properties, for example, a different pattern of delays, population coverage, weekly or seasonal cycles. The dynamics observed in these streams have to be carefully evaluated, and their interpretation might depend on our knowledge of the participating population (for example, monitoring disjoint portions of the same population might provide seemingly contrary results). With an increase in the diversity of prospective disease markers and a relative ease in collecting such information electronically through blogs, mailing lists, feeds, and queries,^19^ a diligent control for data quality and trustworthiness has to be established. In fact, these systems are likely to be sensitive to factors that modify human behaviour but are unrelated to true disease incidence. Unconfirmed data streams can be prone to false alarms because of our limited knowledge on proper signal cali brations.^20^ To understand the interrelationship among various data streams and data sources, it is essential to have an automated support system with auxiliary information related to the population at risk and to spatio-temporal patterns of disease spread. Such information might include demographic profiles and dynamics, population migration and travel patterns, vaccination coverage and herd immunity, calendar of social activities and anticipated mass gatherings. Availability of this information can contribute to the ability to detect an imminent outbreak early (see Table 1 for more examples).

### Overview of modern and cutting-edge methods

Every outbreak is initiated by the introduction of a pathogen to a susceptible population, and represents a unique sequence of elementary events in time and space forming a unique signature, which depends on a multitude of factors. Each outbreak can be characterized by the duration, magnitude, and shape of an epidemic curve. Early detection implies that it is possible to detect a change at the initial stages of an outbreak as it progresses; in other words, to detect an expectation of change. Analytical approaches that address this task can be framed as detection of aberrations at various temporal or spatial or spatio-temporal scales.

The detection of aberrationsFchanges in the current disease distribution as compared with historical or baseline information that may include trend, impulse, spike, and step shiftsFis based on understanding and on the ability to measure the signal-to-noise ratio, where noise represents an underlying trend and a ‘signal’ reflects an outbreak signature. In analyses of time series data, representing both noise and a signal, or a composition of a baseline endemic level with embedded epidemic curve, the use of moving averages, adaptive thresholds, cumulative sums, etc. aims to separate a noise from a signal by removing underlying trends of a presumably ‘known form’. For example, in fitting a harmonic regression to a time series of disease counts with well-pronounced seasonal oscillations,^21, 22^ it is assumed that a trajectory can be approximated by a periodic cosine function. Thus, the removal of underlying trends can be advantageous in removing expectations based on preexisting knowledge, and can also be misleading if the ‘known form’ is chosen incorrectly. In reality, the rationale for the removal of expectation might be questionable: just because we expect a specific pattern does not mean our algorithms should not detect it as an aberration or an outbreak. We may be compromising our algorithms’ sensitivity arbitrarily, when instead we should be using human decision making to complement the system, and know when to adjust for or throw away an ‘expected pattern’. This is similar to decision making in expert-assisted reading of imagery, pattern recognition, and interpretation in medical diagnostics, or object identification in security screening (for example, see Levy and Valleron^23^.

Early detection implies an ability to shorten notification time for an outbreak before extensive damage is done. The more rapid the detection goals, the shorter the temporal scale of data collection and reporting required to support those goals. This implies that weekly or daily data itself may soon be insufficient for desired detection and response, requiring instead hourly reporting. In replacing the surveillance standard of weekly reporting with a shorter response time, a wide range of factors have to be taken into account. Dealing with daily reporting requires an understanding of the effects that the day of the week, scheduled social activities, and holidays might have on the expected temporal pattern. For example, depending on the type of hospital admissionFemergency, in-patient, or outpatient careFthe effect of the day of the week on baseline disease patterns can be different. Thus, an analysis of in-patient and outpatient data streams has to be supplemented with an assessment of timing for provisional updates, population mobility, instantaneous effects of the media on seeking medical care behavioural patterns, etc.

Many existing and widely implemented analytical methods for assessing temporal trends, seasonal patterns, and non-periodic aberrations are based on historical records: the longer the monitoring is in place, the faster an unusual pattern can be detected.^24, 25^ For example, diseases that are relatively frequently observed and have a long and wellestablished history of monitoring, such as illness caused by Salmonella and Campylobacter bacteria, are good candidates for traditional detection techniques that use historical limits methods or moving-window methods.^26^ In such situations, traditional approaches are appropriate and can produce reliable predictions. However, the detection of events that are rare or have never been observed poses a challenge for model specification. Most existing models rely on a gradual change in numbers of observed events and are not adapted for data exhibiting a sudden spike in case numbers at an unexpected point in time.^27^ The nature of emerging health threats requires more sophisticated methods that are sensitive to rapid change and capable of taking into account specific features of an anticipated outbreak (for example, see Martinez-Beneito et al.^28^).

The ability to recognize clusters of events of public health concern adds a valuable dimension to early outbreak detection. New methods that enable spatial scanning for potential clusters that are well tailored to the effective detection of localized events are ready for integration into surveillance systems.^29^ The practical implementation of these methods boosts the demand for better-quality georeferenced data on events of interest.^30, 31^ Where did an exposure occur? Where did symptoms first manifest? Where was health care provided? How were these important spatial identifiers collected, stored, shared, and interpreted? Answers to these questions have become increasingly important. In many surveillance systems, the location of a patient's residence is often used as a proxy for exposure and transmission, which is often a wrong assumption. In instances in which detailed geo-referenced information is available, traditional analytical tools have a limited capacity to use such information effectively, as many of them were developed before complex interlinked geographical information could be routinely collected. New methods have to take into consideration non-euclidean topologies of an outbreak signature. Such topologies are very likely to be observed in spatio-temporal patterns of disease when transmission is amplified by transit, for example, in air travel connecting hubs at non-geographically adjacent places. Complex spatial patterns of geo-referenced exposure–transmission–detection pathways have to be better understood to guide the design of adaptive surveillance systems. Novel techniques such as dynamic mapping,^32^ multivariate visualization, flow mapping,^33, 34^ outbreak signature forecasting,^35, 36^ and large-scale simulations of infection spread^37–39^ have the potential to shed light on the complexity of spatial disease clustering.

### Ideas for the future

**Figure 1:**
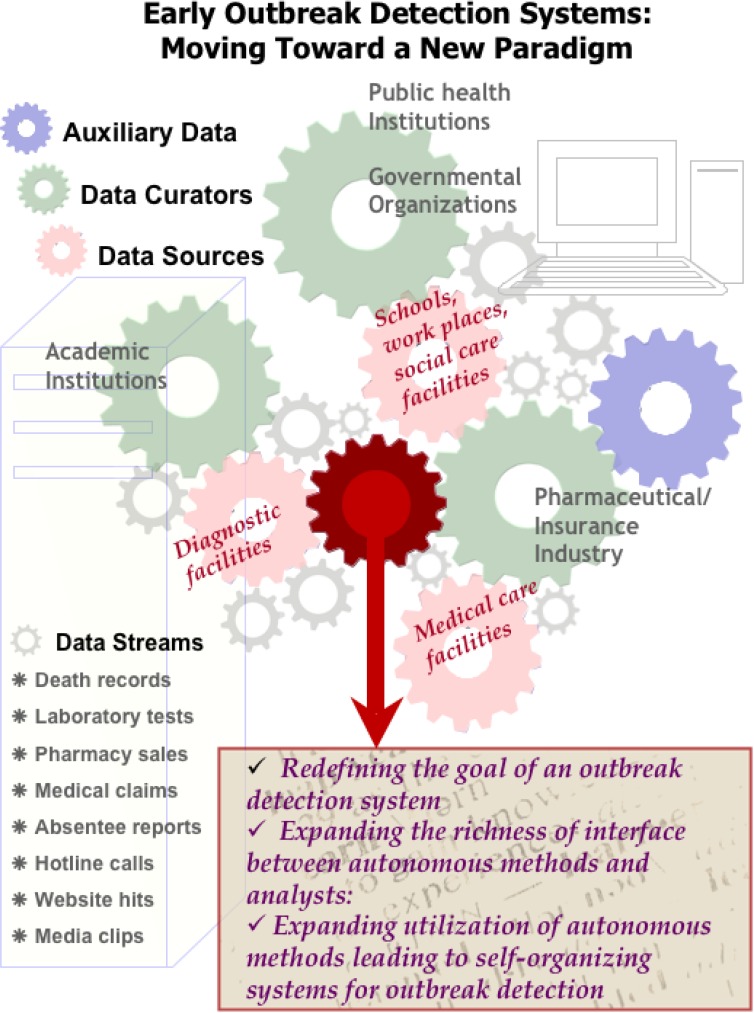
The schematic view of early outbreak detection.

Although there is a plethora of opportunities to expand and refine existing methods, and to develop new ones in completely novel directions as yet unconsidered, we believe that the future of outbreak detection will (at least in the short term) take three very exciting and interrelated paths (see Figure 1).

### Redefining the goal of an outbreak detection system

Although the traditional meaning of ‘outbreak detection’ involves only the detection of incipient ‘greater than normal’ disease incidence, we believe that it may prove vastly important to redefine our target for detection.^40^ Recent advances in the science of risk assessment (We refer the readers to two prominent targeted journals in the field: *International Journal of Risk Assessment and Management* (http://www.inderscience.com/browse/index.php?journalCODE=ijram) and *Risk Analysis* (http://www.wiley.com/bw/journal.asp?ref=0272-4332), which cover various issues of risk detection.) have led to the understanding that comprehensive plans to ensure public safety are the result of characterizing both the probability of an event and the severity of the outcome should the event occur. We propose that there may be benefit in adapting this insight to the field of biosurveillance.

This perspective provides two possible paths for fine-tuning our surveillance efforts: (a) likelihood of event: It may be that particular outbreaks are always introduced through the same pathways. In that case, we may benefit by enhancing the sensitivity of our surveillance in areas in which we know these pathways exist (for example, it may make sense to monitor regions with particular native animals for certain zoonotic infections more closely); and (b) potential result: It may be that we would benefit by focusing on particular populations (for example, the immunocompromised or elderly) for which there is heightened concern based on a lack of herd immunity, the potential for extreme adverse outcomes, or other potentially compromising characteristics. This perspective has been already incorporated into public health planning, but has not yet been incorporated directly into surveillance methods. However, more intriguingly, we can push this idea even further by sliding the sensitivity of our outbreak detection algorithms to reflect areas in which we believe that curtailing an outbreak may be most effective if we identify it sufficiently rapidly. This may even come at the expense of sensitivity in areas in which we understand that we will never have enough advanced warning to effectively alter the course of an epidemic, and could lead to vastly different but more practical and efficient systems. Rather than curtailing the transmission of disease in certain areas, we may aim instead to improve the capability of providing sufficient medical care for all those affected (for example, surge capacity). The possibilities are vast and unexplored. We believe that this more complex goal for outbreak detection algorithms may lead to very different surveillance systems from those already in use.

### Expanding the richness of interface between autonomous methods and analysts

Traditional tools of outbreak detection have been designed to operate in two separate stagesFwith an automatic, computational component in which red flags are raised in the case of a coming outbreak, followed by a distinct humananalyst component in which a person considers the recommendation of the machine and responds by enacting public health responses or dismissing the particular flag as being of low priority or concern. However, we believe that the most effective systems will integrate the input of computational tools with that of human decision makers. Autonomous components of a surveillance system could constantly analyse and organize vast quantities of otherwise overwhelming data, and present the results to a human analyst in ways that may highlight possible anomalies, allowing the analyst to decide which of these directions may be most valuable to pursue. Novel methods for such tools are currently under development within the newly rising field of visual analytics (http://nvac.pnl.gov/, http://www.purdue.edu/discoverypark/vaccine/research/vaat.php),16,30 and we eagerly anticipate further developments in these areas. We believe that these methods will provide drastically more effective tools for biosurveillance.

### Expanding the roles of autonomous methods leading to truly self-organizing systems for outbreak detection

Many of the complications inherent in determining an a priori, human understanding-based definition of ‘outbreak’ may, in fact, be irrelevant. As with many fields of data mining, we believe that there may be vast, untapped potential in the idea of using historical data, not to set thresholds (whether static or dynamic), the violation of which would constitute an outbreak, but instead as training data, that is, data used for sensitivity calibration by a detection algorithm. By introducing concepts of machine learning, it may be that we can allow software to decide where the most useful signals of incipient outbreaks lie in the available data. This can be carried out by providing only the data and points in time during which human analysts concluded ‘manually’ that outbreak conditions were present. Using these methods may lead to surprising and counterintuitive definitions of an outbreak (perhaps having nothing to do with any signal of understood biological relevance). Yet, for practical purposes, it would be unlikely to matter, so long as the detection algorithm arrived at by the autonomous system provided consistent, sensitive, specific warnings of coming outbreaks.

Furthermore, the exploration of autonomous systems need not lie only in the definition of an outbreak itself. As the heterogeneity of data sources increases, it may be that a similarly automated dynamic weighting method for assessing the relative contribution to overall signal strength from each data source could lead to a far more sophisticated and sensitive system for outbreak detection. Again, along the same lines, the underlying topology of data sources could be autonomously explored, allowing clustering algorithms to be applied over different configurations of both time and space. These methods could reveal previously concealed travel patterns, or mechanisms of contaminationFor even perhaps cultural similarities that enable enhanced transmission of an infectious agent. Each of these potentially critical facets, contributing to the spread of infection, would need to be understood and defined manually under the current detection systems; however, by allowing dynamic methods of ongoing machine learning to operate, we may be able to enhance our ability to detect outbreaks while potentially furthering our understanding of the contributing factors driving the disease dynamics. A fully autonomous system, allowed to learn by continual, dynamic exploration of the complete space of incoming surveillance data, could be a prototype for a very powerful self-organizing surveillance system. (For more information on ‘self-organizing systems’, see Levin^41^.)

## Conclusion

We believe that modern advances in assembling data, techniques for collating and processing information for meaningful analysis, and technology to enable integrated analysis and surveillance will support a new paradigm in outbreak definition and detection. We anticipate that these perspectives will provide the highly desired sensitivity and specificity in early detection required by the emerging challenges of global disease surveillance. Although foundational research in these areas is already underway, now that data access and computational power are improving, we believe that the next five to 10 years will be very exciting as these systems are implemented and brought to fruition for practical use.
